# Artificial intelligence to improve polyp detection and screening time in colon capsule endoscopy

**DOI:** 10.3389/fmed.2022.1000726

**Published:** 2022-10-13

**Authors:** Pere Gilabert, Jordi Vitrià, Pablo Laiz, Carolina Malagelada, Angus Watson, Hagen Wenzek, Santi Segui

**Affiliations:** ^1^Departament de Matemàtiques i Informàtica, Universitat de Barcelona, Barcelona, Spain; ^2^Digestive System Research Unit, University Hospital Vall d'Hebron, Barcelona, Spain; ^3^Department of Medicine, Universitat Autònoma de Barcelona, Barcelona, Spain; ^4^Department of Colorectal Surgery, Raigmore Hospital, NHS Highland, Inverness, United Kingdom; ^5^CorporateHealth International ApS, Odense, Denmark

**Keywords:** colon capsule endoscopy, artificial intelligence, screening time, polyp detection, colorectal cancer prevention

## Abstract

Colon Capsule Endoscopy (CCE) is a minimally invasive procedure which is increasingly being used as an alternative to conventional colonoscopy. Videos recorded by the capsule cameras are long and require one or more experts' time to review and identify polyps or other potential intestinal problems that can lead to major health issues. We developed and tested a multi-platform web application, AI-Tool, which embeds a Convolution Neural Network (CNN) to help CCE reviewers. With the help of artificial intelligence, AI-Tool is able to detect images with high probability of containing a polyp and prioritize them during the reviewing process. With the collaboration of 3 experts that reviewed 18 videos, we compared the classical linear review method using RAPID Reader Software v9.0 and the new software we present. Applying the new strategy, reviewing time was reduced by a factor of 6 and polyp detection sensitivity was increased from 81.08 to 87.80%.

## Introduction

Colorectal cancer is the third most common type of cancer worldwide and ranks second on the list of most aggressive and deadly cancers (1). According to the Global Cancer Observatory, out of an estimated total of 1.9 million cases in 2020, this disease has caused the death of more than 935,000 people worldwide (1). This type of cancer has also been linked to unhealthy lifestyle habits such as smoking, alcohol consumption, unhealthy diets or sedentary lifestyle (2, 3). A clear relationship has been drawn between colon cancer and obesity (4, 5) along with genetic predisposition (6).

One of the initial signs of the development of colon cancer is the appearance of polyps in the colon that grow in an uncontrolled manner (7). The detection of polyps when they are still small is crucial to prevent their transformation into cancer. Screening programs are aimed to detect early-stage cancer, improving the patient's chances of survival (8, 9). With the disruption of colonoscopy screening programs due to the COVID-19 pandemic, an increase in incidence of 0.2–0.9% and deaths of 0.6–1.6% is predicted over the next 30 years (2020–2050) (10). To be able to mitigate these effects, well-resourced screening programs are urgently needed. Unfortunately, not all healthcare systems can afford a large increase in demand for colonoscopy which is usually a primary or secondary screening test. Therefore, other equally effective methods should be considered (10, 11). Among those alternatives, CCE has proven to be one of the most safe and effective tools, with an accuracy very similar to that of traditional colonoscopies (12). It is less invasive for the patient (13), it does not cause discomfort during the procedure [only minimal discomfort before the procedure due to required bowel preparation (13)] and no anesthesia is needed. However, the reading of CCE videos is time-consuming and requires qualified medical personnel (14–16). Further studies have compared CCE sensitivity and reading time using different RAPID Reader viewing modes such as QuickView (17–19), as well reading performance of expert and non-expert physicians (20). For CCE to be considered as an alternative procedure in colorectal cancer detection, the use of AI could streamline the process without compromising accuracy (21–23).

AI has been extensively applied to medical imaging problems (24). In the colon capsule endoscopy field, multiple methods have been presented to automatically detect ulcers (25), polyps (26, 27), Crohn's disease (28), bowel cleanliness (29) or blood and mucosal lesions (30). These methods have shown promising results, but they are generally validated with limited data or biased datasets which does not guarantee a good generalization in clinical practice (22, 31). To our knowledge, few attempts have been presented with similar evaluation to the one we propose in this paper. In Aoki et al. (32) a CNN-based method is presented to detect erosions and ulcerations. In Beg et al. (33), a method to detect colonic lesions is introduced comparing two reviewing procedures. There is no doubt that AI has potential benefits to both doctors and patients, but its application to the clinical practice is challenging. AI is not yet at a point where it can completely replace the intervention by human experts (22). Although U.S. Food and Drug Administration (FDA) has approved some assistance algorithms (34), no guidelines establishing the role of AI currently exist. In order to achieve this, these systems would need to gain confidence of medical experts and ethical and regulatory issues would need to be solved (15, 35). This is why it is important to develop systems that cooperate with the experts, facilitating their decision making.

In this paper, we present a novel CNN-based system, AI-Tool, to assist physicians with the detection of colonic polyps. Given a video, it outputs a probability score per image frame to contain a polyp and a heatmap explaining the reasoning of the prediction. This heatmap, allows the expert to focus on the area of the image where the CNN suggests the polyp to be located. An experiment performed with 18 videos revised by 3 expert readers, has shown that the proposed system reduces screening time significantly while it also increases the sensitivity of polyp detection.

## Methods

### Study population

Eighteen videos of patients with at least one colon polyp obtained using the PillCam COLON 2 capsule (Medtronic) were randomly selected for this experiment following a Simple Randomization strategy. The data used in this study are retrospective CCE videos from patients that were conducted on behalf of the NHS Highland Raigmore Hospital in Inverness. All patients from this study came from referrals for symptoms or were on surveillance lists within the Highlands and Islands area of Scotland and had a positive Fecal Immunochemical Test (FIT). Referrals and final diagnoses were made based on local considerations, outside the influence of the teams conducting CCE procedures. Bowel preparation in accordance with a standardized, PEG-based, split-dose cleansing protocol was performed in all patients. All videos were obtained using PillCam COLON 2 which has two heads (front and rear). They were anonymized to protect patient information. Patients' mean age was 58.1 ± 18.7 years (range, 18–92 years) and mean colon transit time was 4 h 10 min (range, 0.17–14.2 h).

Before the experiments began, the two videos obtained from each of the heads of the capsule were meticulously reviewed by four independent CCE readers, experts from now on, in order to create the ground truth for the experiments (gold standard). Each detected polyp was assigned a unique identifier, the timestamp of the first and the last image where the polyp was visible, and from which head it was reported. The independent analysis of the experts was then shared with all experts, reaching a consensus in case of discrepancies. As experts, we have arbitrarily considered CCE readers with at least 3 months of experience in CCE. All of them have formal training in reviewing CCE videos and they analyze about 5–20 videos a week. On a daily basis, they follow the standard review protocol to ensure that each video is reviewed in a consistent, repeatable and well-documented manner. The results of all of them were validated by a medical doctor with 2 years of experience who created a final report about the results. In no case were any concerns reported back by that clinician either from the review nor from any possibly follow-up procedure about the quality of the report. Both, the final report and the gold standard used as ground-truth for the experiment in this study are the responsibility of the medical doctor that approved the results.

During this process, a total of 52 unique polyps were found. The video with the most polyps had 7, while there were 5 videos with only one polyp. The polyps' size was estimated with RAPID. A total of 23 polyps were identified as large (≥6 mm) and 29 polyps as small (<6 mm). There were 5 polyps larger than 10 mm and only one polyp smaller than 3 mm. The characterization of morphologies was done in accordance with the requirements from the referring clinicians, matching standard Paris classifications where possible. In no case polyps were selected or discarded based on size or morphology, all polyps reported by the physicians were included in the study.

### Experimental design

Three experienced CCE readers reviewed the videos selected for this experiment. Each reader reviewed half of the videos using the standard RAPID Reader Software v9.0 (Medtronic) and the other half using the AI-Tool. Results obtained by the experts using each of the tools are reported in terms of number of polyps detected (sensitivity) as well as time needed to complete the reviews (screening time).

The experiment conducted in this study was restricted to the images of the colon. Identification of the entrance and exit of the colon was previously provided to the readers. When they used RAPID software, they were asked to perform the standard screening procedure without any screening time limitation. No information other than a single video identifier was provided to the reviewers during the analysis of the videos. For the AI-Tool, the review time was limited to 30 min regardless of the video's length.

For both tools, readers were required to review the videos without pauses or external stimuli that could lead to distractions. During the review, the readers labeled the images that they identified as a polyp using the tools provided within each of the applications. In the case of RAPID, experts were asked to tag all the unique polyps they found. When using the AI-Tool, experts were asked to make a decision, *polyp, clear* or *other*, for each sequence that was presented to them.

### CCE readers

The expert readers are endoscopy nurses with at least 2 years of experience with CCE. They have a formal CCE training and conduct between 5 and 20 video analyses per week. On a daily basis, they follow a standard operating procedure to ensure that each video is analyzed in a consistent, repeatable way and documented according to common standards. None of these three readers were part of the gold standard creation process.

### Screening tool: RAPID reader v9.0

**Figure 1:**
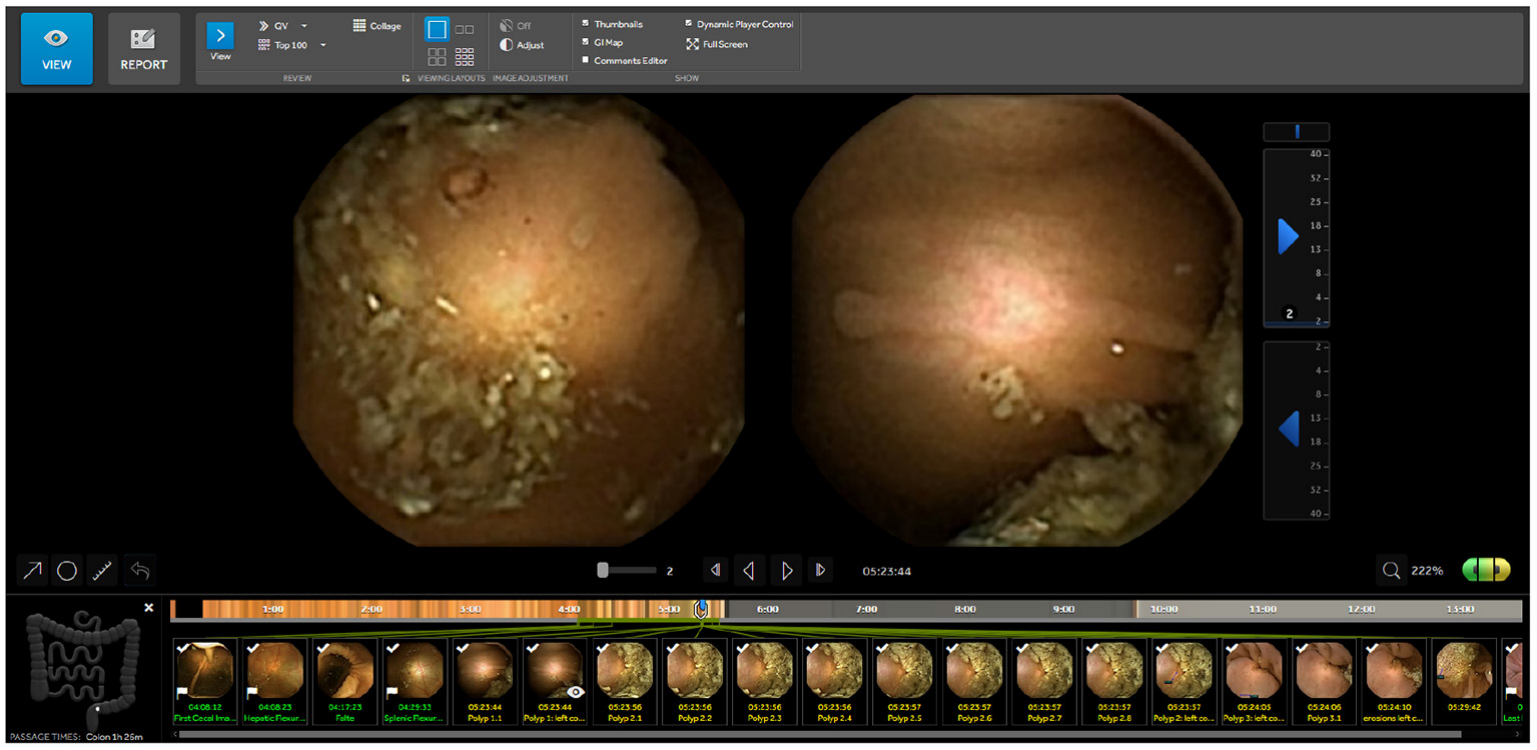
RAPID Reader Software v9.0^*a*^: screen with images from both camera heads (green/yellow) and marked thumbnails. ^*a*^https://www.medtronic.com/covidien/en-gb/products/capsule-endoscopy/pillcam-software.html.

RAPID Reader v9.0 is a Medtronic proprietary software, used to review and interpret wireless capsule endoscopy videos. This software is widely used by colon capsule endoscopy professionals designed to review the video in a comfortable way. The video is shown in temporal order and the app allows to label images, to add comments to images and to measure any part of the image with an integrated ruler function. The user can change the replay speed of the video at any time and the application shows the approximate area of the abdomen where the capsule is located. It also warns the user if the capsule is passing through an area at high speed, so the user can know if special attention is required. RAPID has several modes to review videos: single view with the frontal camera, twin head mode showing both cameras simultaneously (Figure 1) and collage mode which shows a batch of frames selected by traditional computer vision techniques.

### Screening tool: AI-Tool

The AI-Tool is a software designed to assist clinicians in the detection of polyps, by complementing any proprietary video reviewing software, such as RAPID. It embeds a Convolutional Neural Network (CNN) into a web tool that presents images with potential polyps to the user in a sequence of declining certainty. Therefore, images that are very likely to contain a polyp will appear first. At the time we started the study, the CNN from Laiz et al. (26) was the state of the art for polyp detection so it was chosen among other candidates (36–38) as the core of the AI-Tool. The hyperparameters of the model were fixed after a 5-Fold cross validation process using 120 CCE videos (2,080 polyp images and 246 k negative images). Different hyperparameters were tested (the same for the five models) and those that gave the best results in the validation sets were selected. A single model was then trained using the 120 CCE and embedded into the AI-Tool. The experimental validation of the network has shown a sensitivity over 90% at a specificity of 95% when evaluated in a fully automatic setting (when no expert is involved) using full videos. All 120 videos used in the training of the CNN were excluded for this experiment.

The AI-Tool computes two outputs using a CNN: a probability score per frame to contain a polyp and a heatmap to visualize the reasoning behind the score using CAM (39), an algorithm that uses the values of the latest CNN layers to display the image areas most relevant for classification. In this particular case, this method presents the most relevant image zones that allow the CNN to classify an image as polyp.

**Figure 2:**
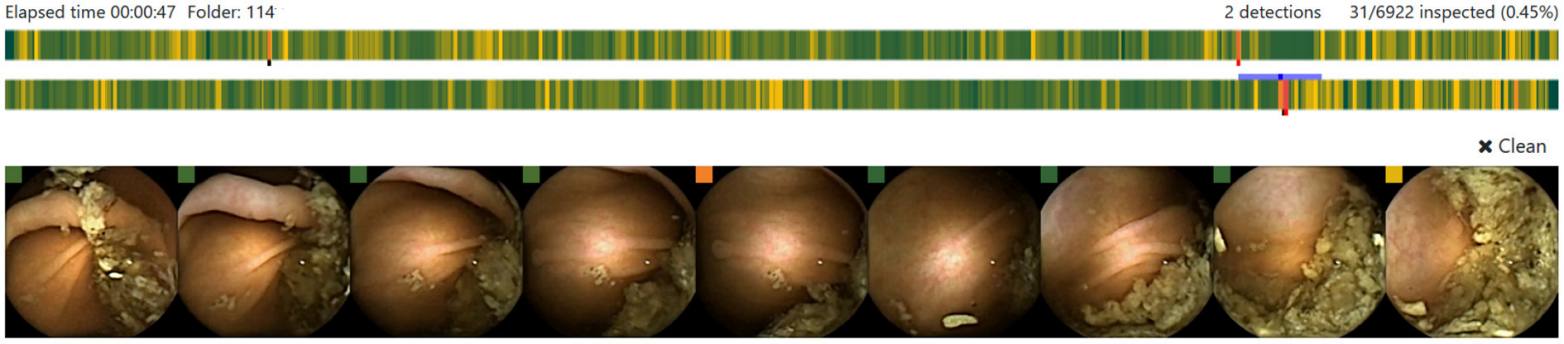
Candidate polyp sequence displayed in the AI-Tool. Each colored bar shows the probabilities for a polyp in one head of the capsule. The proposed image is presented in the center frame and 4 context images are placed by each side.

Each potential polyp image is displayed along with eight context frames, the four preceding and the four following it (Figure 2). For each frame, a colored square is shown to indicate the probability of it being a polyp using a colorblind friendly palette that can be customized when the application starts. Each image can be enlarged by clicking on it, then further information is presented such as the probability or the timestamp of the sequence. The image sequence can be also displayed as a video using the left and right keyboard arrows. Heatmaps can always be activated showing the most likely area to contain a polyp. A further benefit of the heatmap is that it helps readers to understand the reasoning behind the polyp probability and as a consequence, increase their trust in the system.

## Results

### Polyp detection

The overall sensitivity of polyp detection using RAPID as the screening procedure was 81.08% while using the AI-Tool the sensitivity increased to 87.80%. Table 1 shows the percentage of polyps found using both tools distinguishing between three categories: polyp size (in millimeters), visibility (in number of frames) and morphology. The sensitivity using RAPID turned out to be 76.92% for polyps smaller than 6 mm and 85.71% for larger polyps. Both numbers increased when the AI-Tool was used (85.42 and 91.18% for small and large polyps, respectively).

**Table 1:** Detection of polyps distinguishing by size, visibility and morphology.

		**#Polyps**	**RAPID**	**AI-Tool**
Size	Small (<6 mm)	29	76.92%	85.42%
	Large (≥6 mm)	23	85.71%	91.18%
Visibility	Low (<4 frames)	9	58.33%	80.00%
	Normal (4 − 10 frames)	15	73.91%	81.82%
	High (>10 frames)	28	92.31%	93.33%
Morphology	Pedunculated	4	100.00%	100.00%
	Sessile	25	81.82%	92.86%
	Flat	23	75.76%	80.56%
		52	81.08%	87.80%

The biggest difference between both tools was observed in the visibility of the polyp. For polyps appearing in a few frames (low visibility), the table shows a significant improvement when using the AI-Tool. While RAPID achieved an accuracy of 58.33%, the AI-Tool reached 80.00%. This represents an increase of 21.67% in this category. Smaller improvements using the AI-Tool were also reported for polyps appearing in a larger number of frames.

These results show that small polyps and polyps that appear for only a few frames are more likely to be detected using the AI-Tool than using the RAPID application.

### CCE screening time

One of the aims of this study was to compare the time needed for the detection of polyps using RAPID and our AI-Tool. The average time required for the experiments performed with RAPID was 47.11 min (11.6 min for each hour of CCE video reviewed) with a maximum of 126 min. Let us recall that the time for analysis using the AI-Tool was fixed at 30 min for all the experiments.

**Figure 3:**
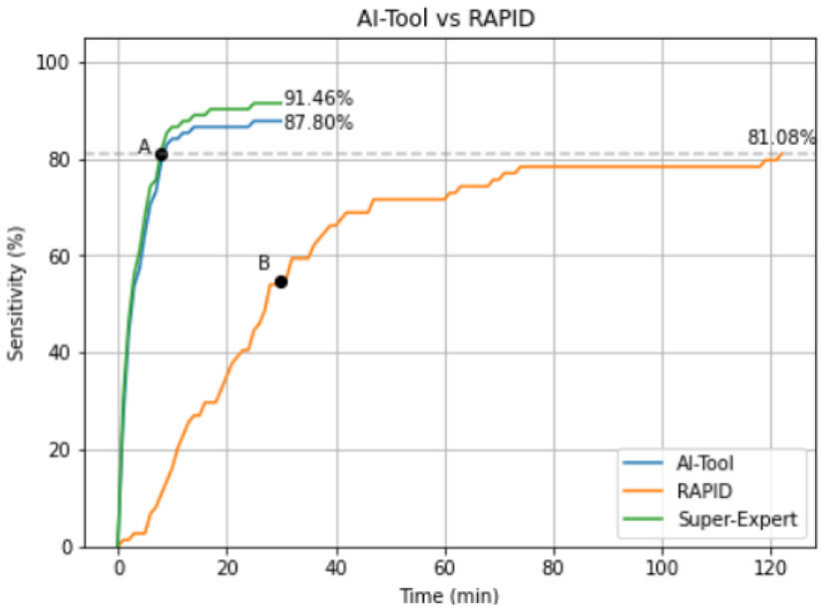
Mean sensitivity curve of the experiments using both applications. In green the Super-Expert curve (gold standard) that represents the maximum value that the blue line could reach. This curve has been calculated simulating an expert who never makes mistakes when identifying a polyp while using the AI-Tool.

Figure 3 shows the average sensitivity curve as a function of time for both applications. AI-Tool took only 8.00 min to reach the same accuracy as RAPID (point A). Since the mean of RAPID experiments was 47.11 min we can state that the AI-Tool reduces the time needed to reach the same accuracy as RAPID by a factor of 47.11/8.00 ≈ 6. We can also see that RAPID experiments reached a 55.41% of sensitivity (point B) by minute 30 when the AI-Tool experiments finished.

The shape of the curves is also an aspect worth considering. While the RAPID curve has an almost linear behavior, the AI-Tool curve shows an initial steep slope and, after minute 16, it is almost flat. In the first 10 min of the analysis 84.14% of the polyps are detected. In the next 10 min, this number rises to 86.59%, which represents an increase of 2.45% in this period. Finally, only 1.21% of the polyps are detected in the last 10 min of the experiment. This indicates that our application is proposing the relevant images in the first minutes of visualization. It is also worth mentioning that without limiting the time to 30 min as we did, the detection of polyps may slightly increase because those with a very low score would have been presented to the reviewers. However, this was not the objective of our study, since we aimed to see if the video review could be done better and in a shorter period of time.

### Qualitative results

**Figure 4:**
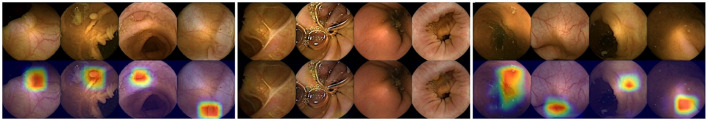
**Left**: Images of correctly identified polyps with their respective heatmaps (True Positives). **Center**: Images of polyps with a very low score (False Negatives). **Right**: Images that do not contain a polyp but still have a very high score (False Positives).

Heatmaps are a key element of our tool. They allow the medical staff to trust the system and make an informed decision on each image sequence. Figure 4 shows the heatmaps activation for three different categories. First, in the image on the left, we see high-scoring polyps. We observe how the heatmaps generated by the CAM algorithm are well defined and show the area where the polyp is located. In the center, we see polyps with a very low score, which the network erroneously classifies as negative. In this case, the heatmaps are not activated. Finally, in the image on the right we can see images that do not contain any polyp but to which the system assigns a high score.

It can also be seen that the false positive patterns correspond to textures and morphologies compatible with polyps. In almost all of them a rounded area is displayed which, without the context of the other images, can be difficult to classify as a polyp image or not. In addition, this is a valuable information for the reviewers of the video, as the heatmap shows the area on which it is important to focus their attention.

**Figure 5:**
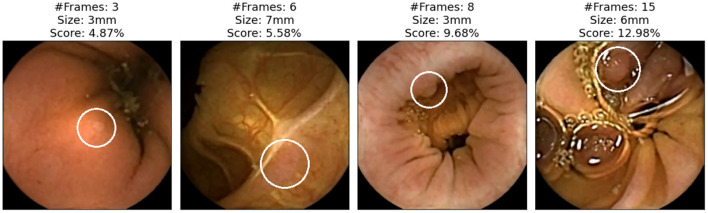
Polyp frames to which the app has attributed a small score and, therefore, none of the experts have been able to review in the first 30 min. Polyps are circled in white.

We now focus on showing the images of polyps that have not been detected with either of the two applications (specificity). Figure 5 shows those polyps not detected with the AI-Tool because of the imposed time restriction (30 min). The score given to these image does not exceed 15%, therefore, the experts never reviewed them. In fact, these four images are the polyps with the lowest score of this study. The polyps of these images are difficult to find since they are partially occluded or do not present a regular morphology.

**Figure 6:**
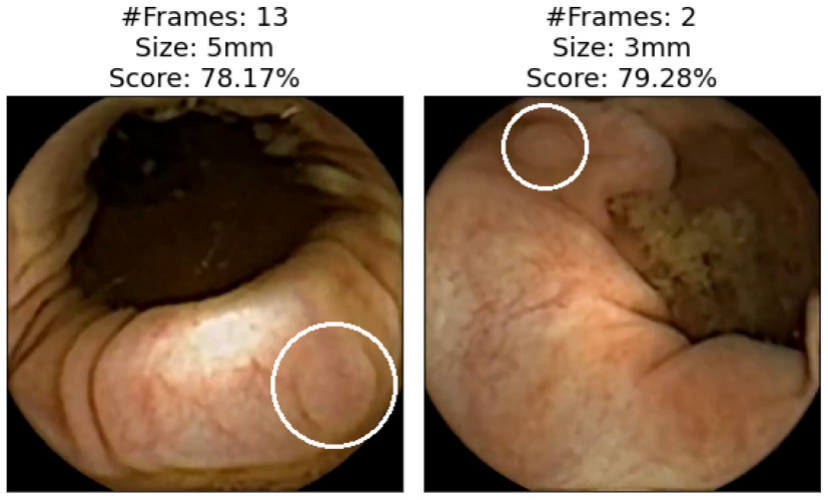
Images that all experts have reviewed and found not to be polyps. Polyps are circled in white.

Figure 6 shows images of polyps reviewed and discarded by all the experts even though the AI-Tool assigned them a remarkably high probability. These missed polyps are the result of human error or discrepancies between the video reviewer and the experts who generated the ground truth.

**Figure 7:**
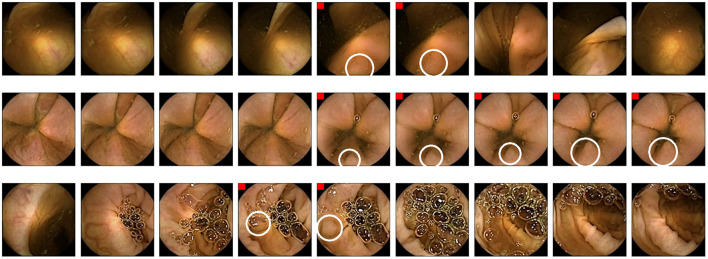
Example of polyps missed in RAPID experiments. Polyp frames are tagged with a red square. Polyps are circled in white.

Finally, Figure 7 shows some examples of polyp images missed in RAPID experiments but correctly detected using the AI-Tool. Due to the size of the polyp and the fast movement of the capsule that took few images, these polyps are especially difficult to find using RAPID. In contrast, they are easy to find for AI-Tool users as they are presented with the clearest image.

## Conclusions

In this paper, thanks to the AI enhancement, we mitigate one of the main drawbacks of CCE, the required time for the analysis while increasing the detection rate of the experts. redWe consider that these improvements further improve the value proposition of CCE as a clinically viable alternative to traditional imaging methods of the gastrointestinal tract.

The proposed method, AI-Tool, uses the output of a CNN architecture. It scores frames based on the probability of containing a polyp and then it reorders the video images to present the most relevant ones first. The validation was performed by three clinical experts that analyzed 18 videos, comparing the standard method with and without the proposed AI-enhanced application. With the assistance of the AI-Tool, the time required to review the videos was reduced by a factor of 6 and the sensitivity increased from 81.08 to 87.80%. In the case of small polyps (<6 mm), the improvement in sensitivity obtained by the AI-Tool was 8.50%, and, for polyps with low visibility (seen in <3 frames), the improvement in detection was 21.67%.

## Data availability statement

The data used during the present study are not publicly available as they are property of National Services Scotland (NHS Highland) but are available through co-author AW (angus.watson@nhs.scot) upon reasonable request.

## Ethics statement

The studies involving human participants were reviewed and approved by University of Barcelona's Bioethics Commission, Institutional Review Board IRB00003099. The patients/participants provided their written informed consent to participate in this study.

## Author contributions

Data collection was performed by HW. The first draft of the manuscript was written by PG. All authors commented on previous versions of the manuscript, study conception, design, read, and approved the final manuscript.

## Funding

This work has been also supported by MINECO Grant RTI2018-095232-B-C21, SGR 1742, Instituto de Salud Carlos III as well as the Innovate UK project 104633.

## Conflict of interest

Author HW is co-founder of CorporateHealth International, a company that may be affected by the research reported in the enclosed manuscript. The remaining authors declare that the research was conducted in the absence of any commercial or financial relationships that could be construed as a potential conflict of interest.

## Publisher's note

All claims expressed in this article are solely those of the authors and do not necessarily represent those of their affiliated organizations, or those of the publisher, the editors and the reviewers. Any product that may be evaluated in this article, or claim that may be made by its manufacturer, is not guaranteed or endorsed by the publisher.
